# Wild Halophytes: Tools for Understanding Salt Tolerance Mechanisms of Plants and for Adapting Agriculture to Climate Change

**DOI:** 10.3390/plants12020221

**Published:** 2023-01-04

**Authors:** Marius-Nicușor Grigore, Oscar Vicente

**Affiliations:** 1 Faculty of Medicine and Biological Sciences, “Ștefan cel Mare” University of Suceava, Str. Universității 13, 720229 Suceava, Romania; 2 Institute for the Conservation and Improvement of Valencian Agrodiversity (COMAV, UPV), Universitat Politècnica de València, Camino de Vera s/n, 46022 Valencia, Spain

**Keywords:** climate emergency, crops’ wild relatives, glycophytes, halophytes, phytoremediation, salt stress, salt tolerance mechanisms

## Abstract

Halophytes, wild plants adapted to highly saline natural environments, represent extremely useful—and, at present, underutilised—experimental systems with which to investigate the mechanisms of salt tolerance in plants at the anatomical, physiological, biochemical and molecular levels. They can also provide biotechnological tools for the genetic improvement of salt tolerance in our conventional crops, such as salt tolerance genes or salt-induced promoters. Furthermore, halophytes may constitute the basis of sustainable ‘saline agriculture’ through commercial cultivation after some breeding to improve agronomic traits. All these issues are relevant in the present context of climate emergency, as soil salinity is—together with drought—the most critical environmental factor in reducing crop yield worldwide. In fact, climate change represents the most serious challenge for agricultural production and food security in the near future. Several of the topics mentioned above—mainly referring to basic studies on salt tolerance mechanisms—are addressed in the articles published within this Special Issue.

## 1. Introduction

The increase in crop yields necessary to feed a growing world population, expected to reach 10 × 10^9^ people by 2050, is seriously hampered in the present global warming scenario. Indeed, climate change constitutes the most critical challenge for agricultural production and food security in the next few decades [1,2,3]. Amongst the multiple environmental stress conditions negatively affecting plant growth and productivity, drought and soil salinity are the major causes of the reduction in crop yields worldwide [4]. Climate change contributes to the increasing loss of cropland because of longer, more frequent and severe drought periods, as well as secondary salinisation, especially in areas cultivated under irrigation schemes in arid and semiarid regions. Some estimations indicate that more than 20% of irrigated farmland is already seriously affected by salinisation, and this figure is expected to increase in the coming years [5,6].

The genetic improvement of crop salt tolerance—by classical breeding or using transgenic or genome editing approaches—appears to be the most sensible strategy with which to address the problem of reduction in yields due to land salinisation (see, for example, [7,8,9]). This approach, in turn, requires elucidating the physiological, biochemical, and molecular mechanisms underlying salt tolerance. Mostly model species, such as *Arabidopsis thaliana* and a few crops, have been used to investigate these mechanisms, even though these species are rather sensitive to salt stress [10]. It is true that salt tolerance depends on basic, conserved responses to salinity—including the control of ion transport and ion homeostasis, osmolyte biosynthesis for osmotic adjustment, and activation of antioxidant systems—which are triggered in all salt-affected plants, tolerant or not [11,12,13]. However, the relative efficiency of these responses and the specific mechanisms of tolerance used vary widely in different species. Therefore, it seems evident that salt-tolerant plants are more appropriate for use as experimental systems for dissecting salt tolerance mechanisms, and that there is no single ‘model’ species that will provide enough information.

In the frame of this Special Issue’s topic, it is important to differentiate between a ‘glycophyte’ and a ‘halophyte’. Most plants, including all major crops, are glycophytes, meaning that they are sensitive to relatively low levels of salt in the soil. Halophytes, on the other hand, are wild plants adapted to saline environments that are able to survive and complete their life cycle in habitats with a soil salinity equivalent to at least 200 mM NaCl, although some can withstand salinities even higher than that of seawater [14,15]. Obviously, this is an ‘operational’ (and useful) definition, but, in fact, plant species show a continuous range of salt tolerance—or sensitivity. Thus, many glycophytes may have different degrees of salt tolerance, whereas there are salt-tolerant plants defined as ‘obligatory’ or ‘facultative’ halophytes. In addition, although salt tolerance depends mainly on the genotype, it is affected by many other factors, such as the plant developmental stage or the simultaneous presence of other stressful conditions. Therefore, the distinction between glycophytes and halophytes is not so clear-cut (see [16] for an extended discussion of these issues).

In any case, halophytes are ideal experimental material for fundamental studies of salt tolerance mechanisms in plants at the physiological, biochemical, and molecular levels. They can also provide biotechnological tools for improving the salt tolerance of conventional crops—for example, salt tolerance genes that could enhance this trait when expressed in transgenic plants, or salt-induced promoters used for the expression of those genes. In addition, some wild halophytes could be commercially cultivated, representing the basis of a sustainable ‘saline agriculture’. This would require previous domestication and some breeding to improve agronomic characteristics, but the point to be highlighted is that they already possess salt tolerance, the most difficult trait to incorporate via conventional breeding. These ‘new’ crops could be grown in salinised land and irrigated with brackish or even seawater, not competing with our conventional crops for limited resources: fertile land and good-quality irrigation water. This Special Issue attempts to cover all the aspects mentioned above regarding halophyte basic research and its applications.

## 2. Special Issue Contents

A suitable strategy by which to elucidate salt tolerance mechanisms is performing comparative analyses of the physiological and biochemical responses to salt stress of genetically related taxa with different degrees of tolerance. The selected genotypes may include glycophytic and halophytic species of the same genus or related genera, or different cultivars, varieties, or accessions of the same species (e.g., [17,18,19]). This approach is used in several papers included in this Special Issue. Thus, Ghanem et al. [20] analysed the responses to increasing salinity of three obligate halophytes, *Arthrocnemum macrostachyum*, *Sarcocornia fruticosa*, and *Salicornia europaea* (Amaranthaceae), collected from the same natural habitat, at the vegetative phase of development, measuring biomass and several biochemical stress markers—ion, chlorophylls, proline and antioxidant compounds contents, and some antioxidant enzyme activities. Based on the obtained data, the authors suggested that the investigated species adopt two differential strategies: salt tolerance in *S. europaea* appears to be primarily due to the activation of antioxidant enzymes and the biosynthesis of proline, whereas in *A. macrostachyum* and *S. fruticosa,* it is based on the rearrangement of the chlorophylls ratio and the biosynthesis of antioxidant compounds (carotenoids, phenolics, and flavonoids). 

Calone et al. [21] subjected plants of the perennial species *Sarcocornia fruticosa* and the annual *Salicornia europaea* and *Salicornia veneta* to 30 days of intense salt stress (700 mM NaCl) and water deficit (complete withholding of irrigation), followed by 15 days of recovery (irrigation with non-saline water). Growth parameters and biochemical stress markers were determined after the stress and recovery treatments. The three species showed high tolerance to salt stress, based on the accumulation of ions (Na^+^, Cl^−^, Ca^2+^) in the shoots and the synthesis of organic osmolytes. Interestingly, active ion transport to the shoots and high levels of glycine betaine were also observed in non-stressed control plants and after the recovery period, suggesting the presence of constitutive stress defence mechanisms. The three halophytes were found to be sensitive to water stress, although *S. fruticosa* to a lesser extent than the two annual species; this could be due to adaptation to a drier habitat than that of the *Salicornia* species, but a more gradual stress-induced senescence in perennials may also contribute to the greater drought tolerance of *S. fruticosa*. 

The article by Ibraheem et al. [22] refers to another genus of the Amaranthaceae family. The authors compared the physiological and metabolic stress responses of three *Suaeda species* (*S. monoica*, *S. vermiculata*, and *S. schimperi*) in their natural saline environments—contrary to the other papers in this Special Issue, which report studies under controlled greenhouse conditions. Therefore, this work required extensive soil analyses to determine not only the salinity level but also other stress factors affecting the plants in the field, such as deficiency in essential nutrients and the presence of toxic heavy metals. Soil characteristics were then correlated with metabolic parameters in the plants—organic and inorganic nutrients, photosynthetic pigments, amino acid profiles, oxidative stress markers, and antioxidant metabolites, amongst others. The results demonstrated common tolerance mechanisms, such as the use of Na^+^ and other inorganic elements as cheap osmotica, as well as species-specific stress responses. In particular, the three *Suaeda* species are promising halophytes for the phytoremediation of heavy metal-contaminated soils, showing some specificity in their capacity to accumulate different heavy metals.

The genus *Plantago* (Plantaginaceae) includes halophytes and glycophytes, as well as drought-tolerant species, and is particularly well suited for investigating plant stress tolerance mechanisms. Ltaeif et al. [23] compared the salt stress responses of two halophytes of the genus, *P. crassifolia* and *P. coronopus,* and two glycophytes, *P. ovata* and *P. afra*. As expected, the biochemical responses were different in the two groups of plants; the halophytes accumulated higher leaf Na^+^ and proline contents and showed a lower level of oxidative stress. It was confirmed that *P. coronopus* and *P. crassifolia* are the most tolerant to salt stress, while *P. afra* is the most sensitive of the four species. *Plantago ovata* could not withstand the strongest salt stress treatment (one month in the presence of 800 mM NaCl); nevertheless, it was shown to also be quite resistant to salt stress, apparently through specific responses that differed from those of the halophytes; they include a weaker salt-induced inhibition of photosynthesis, the accumulation of Cl^−^ to higher concentrations in the leaves, and the activation of K^+^ uptake and transport to the leaves under conditions of high external salinity.

Crop wild relatives, generally more resistant to abiotic stress than their cultivated counterparts, constitute an excellent resource for developing new cultivars with enhanced tolerance [24]. Therefore, it is interesting to determine the tolerance mechanisms of wild species of interest. Jekabsone et al. [25] studied the responses of several wild accessions of *Trifolium fragiferum* (Fabaceae) from natural habitats with different salinity levels to controlled salt treatments compared with a commercial cultivar. The authors reported a decrease in plant biomass and changes in partitioning between different organs with increasing salinity, responses that were genotype-specific. In addition, Na^+^ and Cl^−^ accumulation were organ-specific, whereas responses related to mineral nutrition were both genotype- and organ-specific. In several accessions, salinity stimulated reproductive development. The experiments revealed high intraspecies morphological and physiological variability in the responses of the analysed *T. fragiferum* accessions to salinity, meaning that they can be defined as ‘ecotypes’.

Ishikawa et al. [26], on the other hand, compared cultivated rice (*Oryza sativa*, salt-sensitive) with a wild relative (*Oryza coarctata*, salt-tolerant) (Poaceae), demonstrating that the two species use different strategies to control Na^+^ uptake. At the early stage of the salt stress treatment, wild rice increased its xylem Na^+^ loading for a quick and efficient osmotic adjustment but then maintained shoot Na^+^ contents at non-toxic levels by activating the high-affinity K^+^ transporter HKT1;5 (responsible for xylem Na^+^ unloading) and the tonoplast Na^+^/H^+^ antiporter NHX (for sequestering Na^+^ and K^+^ into root vacuoles). On the contrary, *O. sativa* initially limited Na^+^ uptake and transport to the shoot through the activation of SOS1, the plasma membrane Na^+^/H^+^ antiporter, in the roots. However, cultivated rice failed to maintain this response in the long term because SOS1-mediated Na^+^ exclusion is highly energy-demanding. Therefore, the higher salt tolerance of wild rice seems to rely on efficient Na^+^ sequestration in root vacuoles as opposed to Na^+^ exclusion. 

Similarly, Bigot et al. [27] also compared the salt resistance of a crop, tomato (*Solanum lycopersicum*), and a wild relative, *S. chilense* (Solanaceae), focusing on the reproductive phase, particularly Na localisation in floral organs. Salinity was found to affect reproductive development in the two species, but in different ways. For example, salt stress induced a decrease in the number of inflorescences in both species, but the number of flowers per inflorescence or sepal length was only found to be reduced in cultivated tomatoes. Additionally, the fruit set was not affected by salinity, but fruit size and weight were reduced in *S. lycopersicum*. Growth in the presence of salt decreased the stamen length in *S. chilense*, which was accompanied by a reduction in pollen production and an increase in pollen viability. The work included an extensive analysis of the concentrations and localisation of different ions (Na^+^, K^+^, Mg^2+^, and Ca^2+^) in reproductive structures, which differed in the two studied species. For example, Na^+^ was found to be predominantly located in non-reproductive floral organs in *S. lycopersicum* and in the male floral organs of *S. chilense*. The expression of different genes involved in ion transport, analysed by qRT-PCR, also differed in flowers of both species. This study concludes that *S. chilense* was more tolerant to salinity than *S. lycopersicum* during the reproductive phase, which could be associated, at least in part, with the different distribution and transport of ions in their flower organs.

The article by Cárdenas-Pérez et al. [28] also addresses basic salt tolerance mechanisms, although without involving comparative studies of different species but instead a single one, *Salicornia europaea* (Amaranthaceae). Combining morphological, anatomical, and biochemical analyses and advanced statistical methods, this study found that *S. europaea* grows optimally between 200 and 400 mM NaCl and that growth is limited at 0, 800, and 1000 mM NaCl. Almost all analysed traits were found to be dependent on the salinity level but differently affected. The most affected traits included photosynthetic pigments and protein content, plant surface area, peroxidase activity, and anatomical traits related to cell shape. Although this species has been extensively studied, these results significantly expand the present knowledge on the changes in *S. europaea* functional traits in response to salt stress.

Mir et al. [29] investigated the mechanisms of environmental stress tolerance in the threatened halophyte *Limonium angustebracteatum* (Plumbaginaceae), an endemic species of the east and southeast of the Iberian Peninsula of high conservation interest. The study provides new and interesting data on the ultrastructure of salt glands, typical for halophytic members of the family. In addition, several anatomical, physiological, and biochemical responses were assessed in plants subjected to one month of water deficit (complete lack of irrigation) and salt stress (watering with increasing NaCl concentrations, up to 800 mM). The species is highly tolerant to salt stress, but plant growth was found to be significantly inhibited by severe water stress. Apart from salt secretion through salt glands, its salt tolerance is based on the efficient osmotic adjustment by the accumulation of high concentrations of ions (Na^+^ and Cl^−^ as well as K^+^ and Ca^2+^) and the osmolytes proline (Pro) and glycine betaine (GB) in the leaves. The relatively high leaf concentrations of the four ions and GB (but not Pro) in control plants pointed to the presence of constitutive mechanisms of stress tolerance. A large increase in root K^+^ concentrations; the active transport of Na^+^, Cl^−^, and Ca^2+^ to the leaves; and an increase in leaf GB contents were observed in water-stressed plants. Although the responses to water and salt stress differed, K^+^ homeostasis was shown to be essential for tolerance to both stress treatments.

Bueno and Cordovilla [30] studied the possible effect of different plant growth regulators—phytohormones such as salicylic acid, gibberellins, cytokinins, and auxins, and the polyamine spermidine—added to a hydroponic culture on plants of the halophyte *Plantago coronopus* subsequently subjected to a salt stress treatment (200 mM NaCl), with a focus on the use of this species in saline agriculture. All these plant regulators improved plant growth in the absence of salt, whereas in salt-treated plants, spermidine application was the most effective pre-treatment, inducing the strongest growth stimulation, osmolyte (sorbitol) accumulation, and an increase in antioxidant metabolites (phenolic compounds and flavonoids). The authors conclude that this treatment, activating defence mechanisms against stress, could contribute to improving salt tolerance in *P. coronopus*. 

The paper by Sánchez-Gavilán et al. [31] describes the phytochemical analysis of several Amaranthaceae species of the genera *Sarcocornia* (*S*. *alpini*, *S*. *pruinosa*, and *S*. *perennis*) and *Arthronemum* (*A*. *macrostachyum*) from different coastal and inland salt marshes of the Iberian Peninsula. Separation by gas chromatography or HPLC, coupled with mass spectrometry, was used to identify bioactive compounds (phenolic acids, flavonoids, lipids) in the plant material. Trans-cinnamic, salicylic, veratric, coumaric, and caffeic acids were present in all analysed species, whereas ferulic acid was only detected in *A*. *macrostachyum*. The identified flavonoids were cyanidin-3-*O*-arabinoside, luteolin-7-glucoside, dihydroquercetin, and p-coumaroyl-glucoside. Regarding fatty acids, palmitic, linoleic, and oleic acids were detected in *Sarcocornia* as the most abundant, whereas palmitic, linolenic, and stearic acids were the main fatty acids present in *A. macrostachyum*. Apart from the biological function of these secondary metabolites in the mechanisms of stress tolerance, their properties (e.g., as antioxidants) increase the interest in the use of these species for commercial cultivation in the frame of sustainable saline agriculture because of their high nutritional value.

Finally, the article by Carreiras et al. [32] addressed the question of whether heavy metal preconditioning could influence the salinity tolerance of *Spartina patens* (Poaceae), an invasive halophytic grass that represents a severe problem for the biodiversity of Mediterranean salt marshes. The authors compared the responses of plants from two salt marshes of the Tagus estuary (Portugal), one pristine and the other contaminated by heavy metals, to increasing salinity. The analysis of photochemical processes, photosynthetic pigments profiles, antioxidant enzyme activities, and lipid composition in plants of the two populations revealed intraspecific physiological differences, resulting in the better adaptation and tolerance to salt stress of *S. patents* from the contaminated marsh, especially at high salt concentrations. Those differences include, for example, salt-induced increases in the chlorophyll a/b ratio and oleic acid content in plants from the heavy-metal-contaminated area or the stronger generation of ROS, and therefore more intense plant damage, in the population from the pristine marsh. The article also discusses the implications of this variability at the population level for the frequency and distribution of the species in salt marshes in the face of climate change.

## 3. Conclusions

This Special Issue, covering several topics included in its proposed scope and a wide range of halophytes, comprises 12 published articles. In five of them, the investigated species belong to the Amaranthaceae family—which includes some of the most salt-tolerant taxa known—of the *Arthrocnemum*, *Sarcocornia*, *Salicornia*, and *Suaeda* genera; however, members of the Plantaginaceae, Fabaceae, Poaceae, Solanaceae, and Plumbaginaceae families are also studied in other papers. The selected halophytes include some that have been extensively studied before, such as *Salicornia europaea* or *Plantago coronopus*, as well as a narrow endemic species of high conservation interest (*Limonium angustebracteatum*), or *Spartina patens*, an invasive species that represents a serious risk to the biodiversity of Mediterranean salt marshes. Most of the accepted articles deal with elucidating the mechanisms of salt tolerance, in several cases based on comparative studies of the salt stress responses of related taxa with different degrees of tolerance. Other papers include more applied aspects, such as phytochemical analyses that support the commercial cultivation of some halophytes because of their high contents of healthy antioxidant metabolites or the possibility of using others species in the phytoremediation of heavy metals-contaminated soils. Anatomical, physiological, and biochemical analyses are the most common experimental approaches used in these studies. Altogether, this Special Issue provides a comprehensive and updated overview of the biology of halophytes, contributing to expanding our knowledge of this amazing group of salt-tolerant plants. Other aspects initially included in the scope of the Special Issue, such as studies conducted using molecular biology or ‘omics’ approaches or the generation of biotechnological tools for the breeding of salt tolerance in conventional crops, have not been directly addressed here, but could be included in a possible ‘second edition’ of the Special Issue.
